# The Knowledge, Attitude and Practices of the Caregivers about Oral Health Care, at Centers for Intellectually Disabled, in Southern Region of Saudi Arabia

**DOI:** 10.3390/healthcare8040416

**Published:** 2020-10-21

**Authors:** Shahabe Saquib Abullais, Falah Mohammed Falah Al-Shahrani, Khalaf Mohammed Saeed Al-Gafel, Al-Harthi Abdulrahman Saeed, Shouq Abdulrahman Al-Mathami, Shaeesta Khaleelahmed Bhavikatti, Abdul Ahad Ghaffar Khan

**Affiliations:** 1 Department of Periodontics and Community Dental Sciences, College of Dentistry, King Khalid University, Abha 62529, Saudi Arabia; drshaeesta@gmail.com (S.K.B.); abahkhan@kku.edu.sa (A.A.G.K.); 2 Research Center for Advanced Materials Science, King Khalid University, Abha 62529, Saudi Arabia; 3 College of Dentistry, King Khalid University, Abha 62529, Saudi Arabia; Alhmadflah@gmail.com (F.M.F.A.-S.); K4laf12@hotmail.com (K.M.S.A.-G.); dmy121@hotmail.com (A.-H.A.S.); Shou8.3.m@hotmail.com (S.A.A.-M.); 4 Department of Oral and Maxillofacial Surgery, College of Dentistry, King Khalid University, Abha 62529, Saudi Arabia

**Keywords:** caregivers, knowledge, attitude, practice, special need

## Abstract

Oral health is perhaps the most neglected aspect of healthcare for persons living in rehabilitation centers, compared to the general population. The caregivers play a vital role in administering daily oral care to residents in rehabilitation centers: The aim of the present questionnaire-based study was to evaluate the caregivers’ knowledge, attitude and practices towards oral healthcare at centers for the intellectually disabled in the Southern region of Saudi Arabia (SA). The present study was designed as a cross-sectional one-point time survey. The samples were selected by using a non-probability convenience sampling method. The self-administered closed-ended questionnaire comprised of 22 items divided into four sections, which was designed to elicit the demographic information, awareness of oral care, attitude towards oral health and practice of the oral healthcare regime among the participants. Of the164 participants, 53.0% and 47.0% were male and female, respectively. Most of the participants—54.9%—were between 30–39 years of age. Participants were almost equally distributed between educational groups, and about 87% had more than 2 years of experience. A brief overview showed a fair level of knowledge and attitude among 61.6% and 58.5% of respondents, respectively. Participants with an education level above that of high school showed good knowledge and attitude scores (*p* < 0.05), whereas females showed better attitude scores compared to the males (*p* < 0.05). Respondents mentioned the difficulties they faced while treating the subjects. The special healthcare workers generally had acceptable oral health knowledge and practices. Caregivers who had lower education levels should be advised for training programs that addressed the importance of oral health services.

## 1. Introduction

The oral cavity has been described as “the window to general health.” According to Seymour, statements such as “You cannot have good general health without good oral health” and “The mouth is part of the body” are now considered obvious [1]. The mouth and oral cavity are the focal points for the interaction of the body with the external environment. Speech, chewing, swallowing and the early stages of digestion are all vital physiological functions carried out by oral cavity. Oral health is also a mirror of a person’s psychological identity [2]. It has been recognized for many years that oral health is perhaps the most neglected aspect of healthcare, especially for those living in rehabilitation centers [3]. Therefore, the degree of unmet dental needs for them is far greater than for the general population [4]. Maintaining optimal oral health in physically handicapped and intellectually disabled individuals can be quite challenging as they also have a compromised overall general health [5,6].

It is well known that good oral health can promote general health, self-esteem, social integration and hence, improves the quality of life [7,8]. Prevention of oral diseases in disabled groups is crucial due to the limited availability of resources and meagre access to oral healthcare facilities [6]. There have been many attempts to explain the reasons for this in public health terms. The reduced access to healthcare for this sector of the population has been discussed extensively including, but not limited to, financial, social, and physical barriers. It also bears a direct relationship to mental and physical disability, anxiety and a person’s inability to co-operate [9]. One of the major areas of concern in oral health is the periodontal health, which is immensely affected due to the lack of oral hygiene, occlusal interferences and teeth malalignment, the lack of masticatory function, occlusal trauma, and a soft diet [10]. The importance of oral health is generally ignored or underestimated in centers where residents are intellectually disabled despite their good medical and physical status [11].

It is a well-established fact that oral health education results in the improvement of attitudes towards and knowledge of dental healthcare, especially amongst the caregivers for patients with special needs [12]. These improvements are found to be significant, with better oral healthcare delivery to individuals with special needs [13]. One study showed that the main reason for a lack of visits to the dentist for this population was an assumption, by the caregiver, that “nothing was wrong with the oral health” [14]. It is thus extremely important to alert caregiver’s to the importance of prophylactic measures (an adaptation of food texture to masticatory ability, prevention of aspiration, prevention of infection and endocarditis), as well as therapeutic measures (localized pain, lack of aesthetics, loss of teeth).

Caregivers play a crucial role, both in providing effective care as well as in educating the importance of adequate oral assessments and oral hygiene to residents of rehabilitation centers [15]. Knowledge and attitudes may differ depending on their educational background, their allotted work load and their overall job satisfaction. Therefore, developing an effective oral health promotion strategy in any given community must be based on an in-depth understanding of the unique needs of the population.

To the best of the authors’ knowledge, there are no reported studies in the literature concerning oral health knowledge among caregivers at the centers for the intellectually disabled in the southern province of Saudi Arabia. A simple assessment of knowledge, attitude and practice (behavior) levels may be the first step in identifying the areas of weakness. Thus, this study was aimed to assess the level of the knowledge, attitudes and practices among caregivers with regards to oral health in the Southern province of Saudi Arabia.

## 2. Materials and Methods

The present study was designed as a prospective cross-sectional study targeting the caregivers at three rehabilitation centers for intellectually disabled individuals in the Southern region of Saudi Arabia. The study was conducted between 12 September 2019 and 12 January 2020. The samples were enrolled by using a non-probability convenience sampling method [16]. A total of 205 caregivers (103 male and 102 female) from three centers were enrolled in the present study, of which 164 participants completed the study. The primary data were collected by a self-administered closed-ended questionnaire consisting of questions regarding oral health and oral hygiene.

The research questionnaire and protocol for this study was presented and approved by the local ethics committee at the College of Dentistry, King Khalid University, Saudi Arabia (SRC/ETH/2018-19/110). The inclusion criteria for the study were: (i) permanent employment in the rehabilitation center and voluntary participation in the study, and (ii) caregivers who could read and understand English and Arabic, so that they could answer the questionnaire.

A self-explanatory, anonymous questionnaire comprised of 22 questions, divided into four sections, which was designed to elicit the following information: (i) demographic information of the participants, (ii) knowledge/awareness of oral care (ten questions), (iii) attitude towards oral health (seven questions) and (iv) practice of the oral healthcare regime (five questions).

A pilot survey was carried out among 15 caregivers, equally selected from each center, to increase the credibility of the questionnaire. The pilot samples were excluded from the main samples of the survey. The core objective of pilot survey was to guarantee the validity and reliability of the questionnaire and to incorporate suggestions from the caregivers, to make the questionnaire more comprehensive and understandable. The face and content validity of the questionnaire was thoroughly assessed and evaluated by the professionals in the field of research. Cronbach’s alpha test was employed to evaluate the reliability and internal consistency of the questionnaire [17]. Cronbach’s alpha value 0.832 and 0.80 were recorded for knowledge and attitude segments indicating that the questionnaire had good reliability and was appropriate for the selected samples.

The sample size was determined based on the assumption that roughly about 25% of the caregivers have a poor level of knowledge about oral healthcare in the study area and with the help of following formula:

(1) $$n\;=\;z^{2}\;\frac{\mathrm{pq}}{{\left(\mathrm{me}\right)}^{2}}$$

p = 0.25 (25.0%) (assumed estimate of prevalence of poor level of knowledge),

q = 0.75 (75.0%) (complement of “p”),

z = 1.96 (score at 95% confidence interval),

me = 0.07 (7.0%, margin of error).

n = 1.96^2 ∗ 0.25 ∗ 0.75/(0.07^2) = 147.

Thus, the minimum sample size required according to this formula is 147.

The significance of the survey was verbally explained to the study subjects and written informed consent was acquired before accepting the questionnaire form. The questionnaires were kept anonymous to preserve the identity of the subjects. The questionnaire was randomly distributed among the caregivers during their working hours, while they were given ample time to fill it; meanwhile, the research coordinators were available to answer any queries related to it.

The data on categorical variables were shown as *n* (% of cases) and the data on continuous variables were presented as mean and standard deviation (SD). The inter-group statistical comparison for the distribution of categorical variables is done using Chi-Square test. The inter-group statistical comparison for distribution of means of continuous variables is done using an independent sample t-test for two groups and by an analysis of variance (ANOVA) procedure for more than two groups. The underlying normality assumption was tested before subjecting the study variables to t-test and ANOVA. In the entire study, the *p*-values less than 0.05 were considered to be statistically significant. All hypotheses were formulated using two-tailed alternatives against each null hypothesis (hypothesis of no difference). The entire dataset was statistically analyzed using Statistical Package for Social Sciences (SPSS ver. 21.0, IBM Corporation, USA) for MS Windows.

## 3. Results

**Figure 1:**
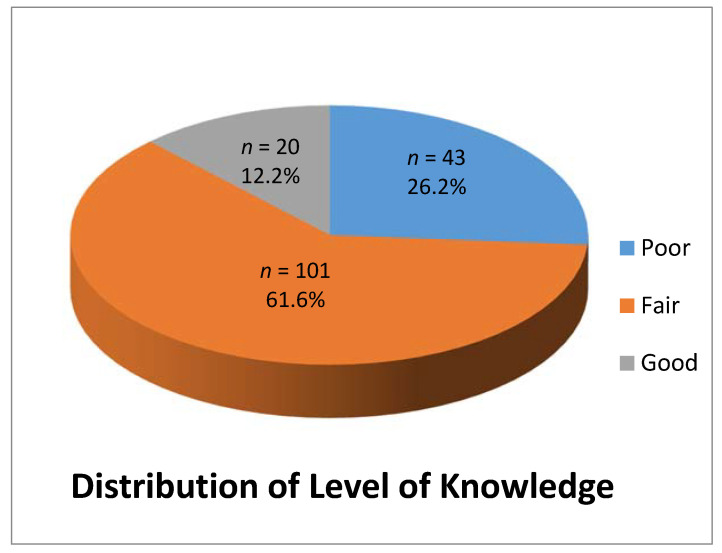
Distribution of level of knowledge among the respondents who participated in the study.

Of the randomly selected sample of 205 caregivers, 164 completed the study. Table 1 represents the detailed distribution of the demographic characteristics of the caregivers who participated in the study. The overall distribution of the level of knowledge among the caregivers showed that 43 caregivers (26.2%) had poor level of knowledge, 101 (61.6%) had a fair level of knowledge and 20 (12.2%) had a good level of knowledge (Figure 1).

### 3.1. Distribution of the Level of Knowledge According to Demographic Characteristics 

The level of knowledge was significantly higher among the caregivers of the younger age group compared to the older age group (*p*-value < 0.05). Caregivers in the 20–29 year age group revealed a significantly good knowledge compared to the other age group (*p*-value < 0.05). The distribution of the level of knowledge based on gender and experience did not differ significantly among the groups (*p*-value > 0.05).

The distribution of the knowledge level differed significantly between the education groups (*p*-value < 0.05). A significantly good level of knowledge was exhibited by the group of caregivers with education above high school level as compared to caregivers with an education below the high school level (*p*-value < 0.05) (Table 2).

**Figure 2:**
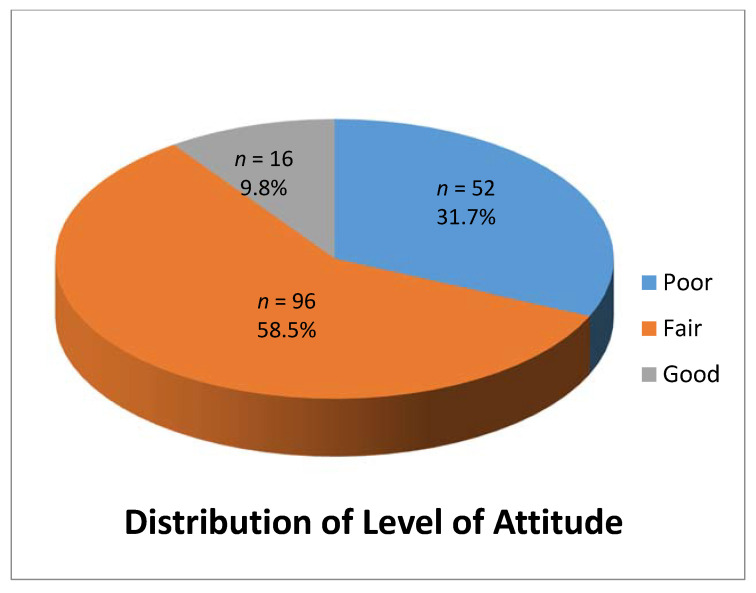
Distribution of level of attitude among the respondents participated in the study.

Overall distribution of the level of attitude among the caregivers showed that 52 caregivers (31.7%) had poor level of attitude, 96 (58.5%) had fair level of attitude and 16 (9.8%) had a good level of attitude (Figure 2).

### 3.2. Distribution of the Level of Attitude According to Demographic Characteristics 

A comparison between the various age groups did not show a significant difference in the level of attitude of the caregivers (*p*-value > 0.05). According to gender, female caregivers revealed a significant fair level of attitude compared to their male counterparts (*p*-value < 0.05). In addition, a significantly good level of attitude was exhibited by the caregivers with an education level above high school as compared to those below high school (*p*-value < 0.05). Whereas, the distribution of the level of attitude did not differ significantly across caregivers with a different duration of experience (*p*-value > 0.05) (Table 3).

### 3.3. Overall Distribution of Responses Related to Practice 

A significantly higher proportion (55.5%) of caregivers replied affirmatively to the question “Do you think that you are performing correct oral hygiene procedures?” (*p*-value < 0.001). Almost half of the caregivers (57.3%) were aware that “2 min, two times a day” is the correct time duration and frequency of tooth brushing (*p*-value < 0.001). In response to the question “How is your rating about the oral care for the residents?” a significantly higher proportion (40.2%) of the caregivers believed it to be difficult (*p*-value < 0.001). A significantly higher proportion (44.5%) of caregivers believed that they would have delivered better oral healthcare if they were given training in the past (*p*-value < 0.001). Almost half (53.7%) of the caregivers selected “Non-cooperative behavior of the residents” as a significant barrier while providing oral healthcare to the residents (*p*-value < 0.001) (Table 4).

**Figure 3:**
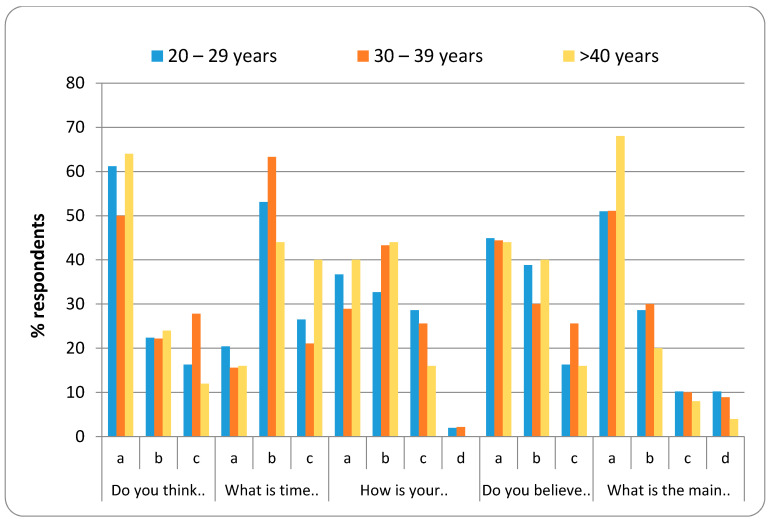
Distribution of responses related to the practice according to age group.

**Figure 4:**
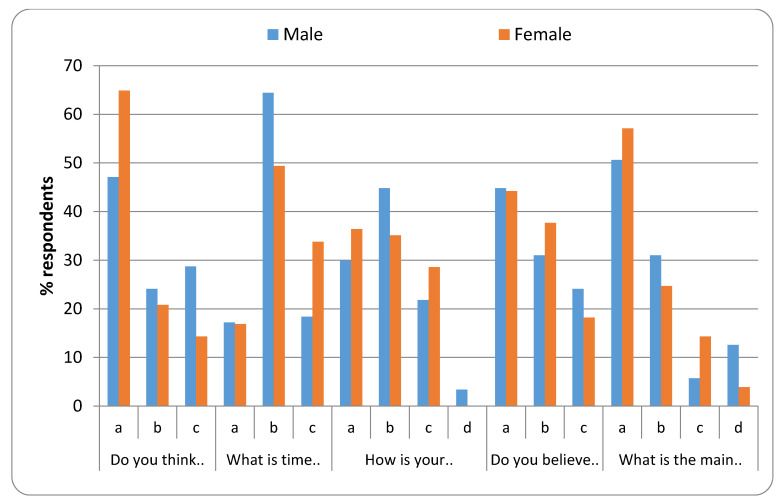
Distribution of responses related to practice according to gender.

**Figure 5:**
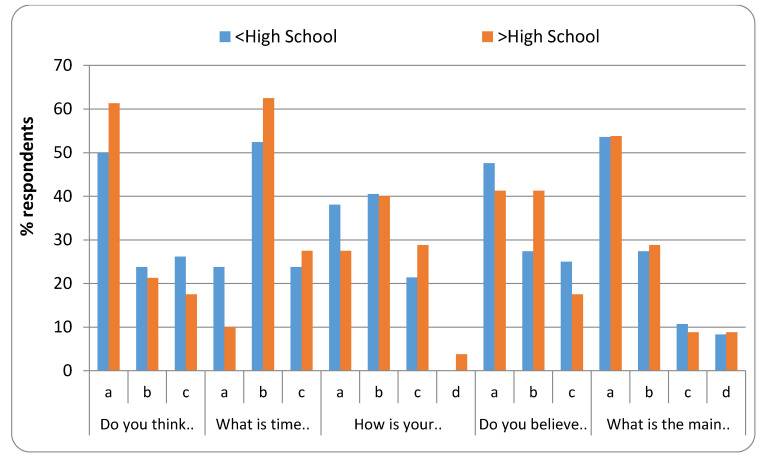
Distribution of responses related to practice according to educational status.

**Figure 6:**
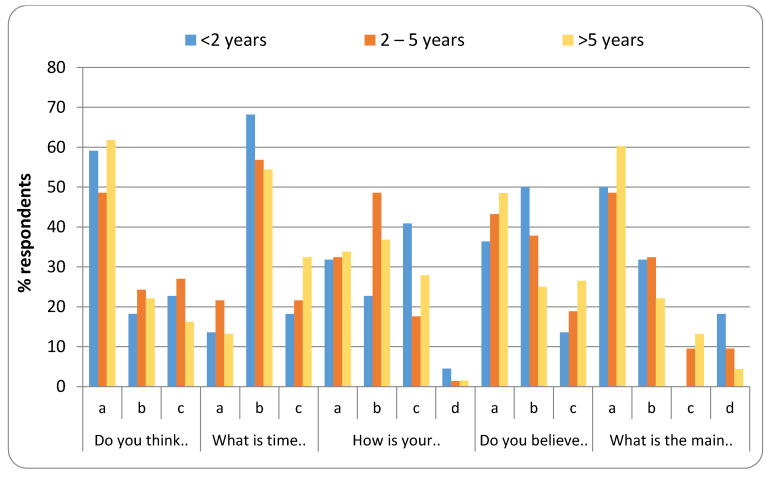
Distribution of responses related to practice according to experience.

Figure 3, Figure 4, Figure 5 and Figure 6 represent the detailed distribution of responses related to practice according to age, gender, educational status and duration of experience of the caregivers, respectively.

## 4. Discussion

A high cavity index and inadequate oral hygiene resulting in gingivitis has been observed in special needs patients. The active role of the dental care team is extremely important in the rehabilitation and oral hygiene maintenance of special needs patients [18]. It has been proved beyond doubt that educational programs to improve oral hygiene have resulted in a significant improvement in the oral health of subjects.

Educational programs related to the dental procedure have also been shown to play a role in the control of associated anxiety and pain. A systematic review underlined that a proper education on dental procedure was demonstrated to have a role in the control of pain and anxiety generally associated with dental procedure [19].

An in-depth knowledge and a positive attitude towards oral health are indispensable tools in providing optimum healthcare. The caregivers’ attitude is a determining factor for the preventive phase of oral care behavior. This positive attitude of the caregiver stems from their embedded knowledge. A good level of knowledge is the encouraging factor for a positive attitude. A good level of knowledge and a positive attitude towards oral healthcare is usually reflected in the behavior and practice of caregivers.

Oral healthcare of patients with special needs seems to be a challenging task for caregivers [20]. This is because people with intellectual disabilities have less favorable outcomes compared to their counterparts as far as oral health is concerned [21,22]. In the present study, a total of 164 caregivers which included 53% of males, completed the study. Most of the caregivers were distributed between 30–39 years of age. However, caregivers between 20–29 years of age had the highest percentage of mean knowledge scores. The distribution of caregivers was almost equal in proportion with respect to the education level either above or below high school. The majority of caregivers possessed more than 2 years of field experience in the disability center.

Knowledge and attitude score was assessed by rewarding “1” point for each correct/positive reply and a score of “0” for each wrong/negative reply. The knowledge and attitude scores for the participants were distributed between range of “0–10” and “0–7” respectively. The final score was presented in the form of a percentage by calculating the sum of all the points followed by computing the percentage. The calculated knowledge and attitude scores in percentage were divided into three subgroups depending on the set criteria: poor knowledge (0–40%), fair knowledge (41–70%) and good knowledge (71% and above) [23].

When results of the mean knowledge and attitude scores were compared according to various demographic characteristics, participants who had an education above the high school level presented the highest percentage of good knowledge (17.5%) and attitude (17.5%) respectively, with a statistically significant difference. Mean attitude scores did not differ significantly across groups representing the different experience and ages of the caregivers. However, pertaining to gender, a significantly higher level of attitude was recorded among male caregivers (12.6%). In general, distribution of mean knowledge scores differed significantly across the various age groups and education, while it did not differ significantly across various groups in terms of the experience and gender of the caregivers.

Interestingly, more than half of the caregivers (55.5%) had the concept of performing correct oral hygiene procedures, which differed significantly with the other caregivers. This was demonstrated by an adequate knowledge of the time and frequency of dental cleaning that the caregivers possessed. As shown in our study, half of them were aware of the correct information on the time and frequency of brushing teeth, as it appears in the professional recommendations. This implied that it is important to brush teeth twice daily for at least 2–3 min [24]. In addition, most of them apparently agreed that better training in oral healthcare would have raised the quality of their dental care. More than a third of the caregivers believed that it was difficult to access the oral care of the residents, and half of them believed that the uncooperative behavior was the main obstacle in treating the residents. However, all demographic characteristics did not affect this behavior. We found that knowledge and attitude scores were significantly lower with caregivers who had an education below high school. This emphasizes that caregivers must be educated to the minimum standards and must have a homogenous orientation. These results are consistent with the study reported by Sumi et al. [25] Another study in a compressive rehabilitation center at Al Kharj, Saudi Arabia by Shah, A. H. et al. showed results that were consistent with the current study, wherein caregivers showed an acceptable awareness regarding the prevention of dental decay by fluoride and also regarding frequency of tooth brushing [26].

In addition, a study conducted by Liu, H. Y et al. [20] in Taiwan showed that the majority of the participants had a positive attitude. Hence this study revealed that education level plays a basic role in a caregiver’s knowledge, wherein caregivers who had a higher level of education showed a greater knowledge of oral health [20]. Another study aimed at assessing the knowledge and attitudes of caregivers in Kuwait showed that the education and attitude of caregivers was strongly associated with the level of knowledge. However, it was weakly associated with practice [27]. Another study conducted by Wyne, A. et al. at Riyadh city in 2014 showed that practices amongst the workers were generally satisfactory, though some weak areas, including a lack of knowledge about fluoridated water, were identified [28].

All potential actions were taken to reduce bias in the current survey, but it was still susceptible to some limitations. The data were recorded as self-reported questionnaires thus it was difficult to avoid question answer errors. Therefore, caregivers may not have provided the actual situation due to social constraints. Non-responsiveness of the study subjects could be a particular concern when the characteristics of non-respondents vary between respondents. Another common limitation could be recall bias, which may have taken place when participants were inquired about past exposure. In addition, this study was conducted in three rehabilitation centers for the intellectually disabled, only in the southern region of Saudi Arabia. The findings of the present survey cannot be generalized to other regions of Saudi Arabia and data should be interpreted with caution and attention. Therefore, more studies in the other parts of Saudi Arabia are anticipated to validate the findings of our study. Our study was targeted at the knowledge, attitude and practices of the caregivers regarding oral healthcare for special need patients with a special emphasis on hygiene education and measures. However, for comprehensive oral care, the caregivers should also be well versed with chronic pain and other implications related to temporomandibular disorders, as it may manifest itself in a wide variety of presentations. Besides, the patients will not be able to express themselves in a conclusive way as to reach the diagnosis. This may open up further research questions in the field.

The present survey recommends that the educational and training programs should be routinely organized by the dental professionals in oral healthcare for caregivers. In addition, the knowledge of dental biofilm and its etiology in dental and periodontal disease should be taught to caregivers. Studies conducted by Wang et al. and Damares et al. reported that such training programs are effective in improving oral health and hygiene conditions among disabled patients [29,30]. According to recommendations by Frenkel et al., an annual reinforcement program should be organized to counter the knowledge fading after a specific period and the turnover of the caregivers in the centers [13].

## 5. Conclusions

Overall, the study explained the often overlooked, yet significant information about the knowledge and attitudes of oral health caregivers for the intellectually disabled patients in the southern region of Saudi Arabia. However, the caregivers’ knowledge and attitude scores concerning oral health were almost satisfactory as compared to the norms with some areas of improvement. In order to improve oral health, caregivers who are less well educated should be advised to participate in training programs on the importance of preventive oral health services and dental treatments. This is important because the parents of children with special needs also look to them for oral healthcare information needed at home. Moreover, preventive techniques concerning oral health must be updated with the continuing advances in research. This will indirectly result in an upgradation of oral healthcare for people with special needs. Finally, researchers should be encouraged to conduct similar studies in this field in other regions of Saudi Arabia in order to promote better oral healthcare for the intellectually disabled.

## Figures and Tables

**Table 1:** Distribution of the demographic characteristics of the respondents who participated in the study.

Characteristics	Category	No. of Respondents	% of Respondents
**Age Group**	20–29 years	49	29.9
	30–39 years	90	54.9
	≥40 years	25	15.2
**Gender**	Male	87	53.0
	Female	77	47.0
**Education**	<High School	84	51.2
	>High School	80	48.8
**Experience**	<2 years	22	13.4
	2–5 years	74	45.1
	>5 years	68	41.5

**Table 2:** Distribution of the level of knowledge according to various demographic characteristics of respondents.

Level of Knowledge										
Characteristics	Category	Poor		Fair		Good		Total		*p*-Value
		***n***	**%**	***n***	**%**	***n***	**%**	***n***	**%**	
**Age Group**	20–29 years	8	16.3	30	61.2	11	22.4	49	100.0	0.050 *
	30–39 years	29	32.2	54	60.0	7	7.8	90	100.0	
	≥40 years	6	24.0	17	68.0	2	8.0	25	100.0	
**Gender**	Male	25	28.7	49	56.3	13	14.9	87	100.0	0.297 ^NS^
	Female	17	22.0	49	63.6	10	12.9	77	100.0	
**Education**	<High School	28	33.3	50	59.5	6	7.1	84	100.0	0.029 *
	>High School	15	18.8	51	63.8	14	17.5	80	100.0	
**Experience**	<2 years	5	22.7	13	59.1	4	18.2	22	100.0	0.900 ^NS^
	2–5 years	19	25.7	46	62.2	9	12.2	74	100.0	
	>5 years	19	27.9	42	61.8	7	10.3	68	100.0	

*p*-value for Chi-Square test. *p*-value < 0.05 is considered to be statistically significant. * *p*-value < 0.05, ^NS^—Statistically non-significant. Higher mean scores indicate a higher level of knowledge and vice-versa.

**Table 3:** Distribution of the levels of attitude according to the demographic characteristics of respondents.

Level of Attitude										
Characteristics	Category	Poor		Fair		Good		Total		*p*-Value
		***n***	**%**	***n***	**%**	***n***	**%**	***n***	**%**	
**Age Group**	20–29 years	18	36.7	26	53.1	5	10.2	49	100.0	0.898 ^NS^
	30–39 years	26	28.9	55	61.1	9	10.0	90	100.0	
	≥40 years	8	32.0	15	60.0	2	8.0	25	100.0	
**Gender**	Male	33	37.9	43	49.4	11	12.6	87	100.0	0.019 *
	Female	20	25.9	50	64.9	7	9.1	77	100.0	
**Education**	<High School	30	35.7	48	57.1	6	7.1	84	100.0	0.035 *
	>High School	20	25.0	46	57.5	14	17.5	80	100.0	
**Experience**	<2 years	8	36.4	13	59.1	1	4.5	22	100.0	0.660 ^NS^
	2–5 years	22	29.7	42	56.8	10	13.5	74	100.0	
	>5 years	22	32.4	41	60.3	5	7.4	68	100.0	

*p*-value for Chi-Square test. *p*-value < 0.05 is considered to be statistically significant. * *p*-value < 0.05, ^NS^—Statistically non-significant. A higher mean score indicates a higher level of knowledge and vice-versa.

**Table 4:** Distribution of responses related to practice among respondents.

Practice Aspects	Response	*n* (%)	*p*-Value
Do you think that you are performing correct oral hygiene procedures?	a Yes	91 (55.5)	0.001 **
	b No	37 (22.6)	
	c Don’t know	36 (22.0)	
What is time duration and frequency of tooth brushing?	a 10 min, two times a day	28 (17.1)	0.001 **
	b 2 min, two times a day	94 (57.3)	
	c 2 min, three times a day	42 (25.6)	
How is your rating about the oral care for the residents?	a Easy	54 (32.9)	0.001 **
	b Difficult	66 (40.2)	
	c Challenging	41 (25.0)	
	d Don’t know	3 (1.8)	
Do you believe that if you were given oral healthcare training, you would be able to practice better oral healthcare?	a Yes	73 (44.5)	0.001 **
	b No	56 (34.1)	
	c Don’t Know	35 (21.3)	
What is the main barrier while treating the residents?	a Non-cooperative behavior	88 (53.7)	0.001 **
	b Language barrier	46 (28.0)	
	c Lack of time	16 (9.8)	
	d Lack of resources	14 (8.5)	

*p*-value < 0.05 is considered to be statistically significant. ** *p*-value < 0.001 (Highly significant).
